# Different approaches to reducing aviation emissions: reviewing the structure-agency debate in climate policy

**DOI:** 10.1007/s44168-022-00001-w

**Published:** 2022-03-07

**Authors:** Nives Dolšak, Aseem Prakash

**Affiliations:** 1 School of Marine and Environmental Affairs, University of Washington, Seattle, USA; 2 Department of Political Science, and Center for Environmental Politics, University of Washington, Seattle, USA

**Keywords:** Airline emissions, Individual responsibility, Tourism, Climate change, Climate-change policy

## Abstract

Aviation emissions account for about 2.5% of global carbon emissions, and by 2050, their share could rise to 22%. This review article explores how climate scholars view the role of structural (policy- or business-focused) or agentic (individual-focused) approaches in reducing these emissions. From a structuralist perspective, aviation emissions require policy changes because they reflect regulatory and business failures to address the climate crisis. By itself, individual actions will not significantly reduce emissions. Moreover, focusing on personal (agentic) action might allow governments and firms to disavow their role in the climate crisis. From an agentic perspective, aviation emissions reflect carbon-intensive lifestyles. Even within the existing policy structures, individuals can reduce the carbon footprint of their travel. At the same time, individuals can serve as influencers, voters, and social movement participants to pressure governments and businesses to develop low-emission air travel policies. Rather than viewing agency and structures as distinctly separate approaches, we suggest that they could co-evolve to create pathways to reduce aviation emissions. Policy initiatives can facilitate individual efforts to reduce air travel emissions, and individual action could shape policies structuring their choices.

## Introduction

Aviation emissions account for about 2.5% of global emissions but drive about 7.2% of global warming due to high-altitude atmospheric effects (Lee et al., 2018). If it were a country, the aviation industry would be the world’s 7th largest carbon emitter. Its emissions are more than twice that of California, which accounts for 1.2% of global emissions (EPA 2019). But for the interruption in air travel in 2020–2021 due to COVID-19, aviation emissions have been growing rapidly. By 2050, their share could rise to 22% of global emissions (EU, 2015).

Aviation emissions have an equity component as well. Because climate change costs are distributed asymmetrically across countries, communities, and generations, equity issues are at the forefront of climate debates (Schlosberg & Collins, 2014; Shue, 2014; Caney, 2014; Dolšak and Prakash, 2022). Aviation emissions epitomize climate inequity: 1% of the world’s population is responsible for 50% of carbon aviation emissions (Gössling and Humpe, 2020; Nicholas, 2021). While individual wealth makes most air travelers immune from the worst consequences of climate change, underprivileged individuals suffer disproportionately from increased frequency and severity of extreme weather events, droughts, new disease vectors, and human displacement (Füssel, 2010; Green, 2016).

The study of the aviation sector provides insights into where climate mitigation actions have been targeted. Scholars favoring structural solutions tend to discount the role of individual action in climate mitigation. Others note the importance of individual agency, which manifests in terms of consumer choices and political action, eventually helping to change the policy framework that structures individual action. In this review essay, we examined two categories of approaches that scholars have proposed to reduce aviation emissions: agentic and structural. We employ a scoping approach (Davis et al., 2015) to survey the literature on approaches to reducing aviation emissions. We employed a two-stage process. In the first stage, we examined climate and environmental policy journals (since 2010) to identify appropriate articles based on a careful reading of the abstracts. After reading the identified articles, we examined their bibliographies to understand where else articles on aviation emissions have been published. We then examined these journals as well. Second, to ensure that we did not miss out on articles published in the journals we had not surveyed, we searched for publications on Google Scholar, using keywords such as “aviation emissions,” “airline emissions,” “flying shame,” “carbon offsets,” “climate action,” “climate mitigation,” “climate movements,” “green purchasing,” “and “agency-structure.” On Google Scholar, we also examined the literature on “social marketing” because climate-inspired consumer choices could be subsumed in the category of pro-social consumer behaviors. Our objective was not to assess whether the agentic or structural perspectives are more dominant in the research or more effective in emission reductions. Instead, our objective was to examine key approaches scholars outline, the strengths and weaknesses of agentic and structural approaches, and how this debate about modes and drivers of climate action speaks to the broader social science inquiry about the role of structure and agency in addressing climate mitigation

The agentic perspective assumes that individuals enjoy some discretion in deciding whether to satisfy their transportation needs via air travel. While individuals might be motivated to take pro-climate actions, they may not have information about aviation’s carbon footprint or low-emission alternatives. If so, information provision could help individuals make superior climate choices. However, scholars note that agentic solutions fail when individuals ignore their climate footprints. But more worrisome, some critics suggest that by taking responsibility for the climate impact of their lifestyles, individuals allow governments and firms to disavow their role in the climate crisis and blame it on individual choices (Mann, 2021). Thus, well-intentioned consumer action provides the cover for regulatory and business failure.

In contrast to the agentic perspective, structuralists suggest that carbon-intensive lifestyles, of which air travel is a prime example, are outcomes of public policies such as fuel subsidies. Moreover, given the scale of the climate crisis and the need for quick action, policy changes are required. Individual choices are also shaped by business-financed advertising (a structural factor) that glorifies social practices, such as travel to exotic destinations, with substantial carbon footprints (Peeters and Dubois, 2010; Lenzen et al., 2018). Because individuals seldom exercise real choices, government and business policy changes are necessary to tackle individual-level, consumption-related emissions.

Structural approaches, particularly the ones focused on governmental policy, can bring about large-scale changes but face political hurdles. Agentic approaches, in contrast, may not work at the same scale but also face fewer political hurdles, given their micro focus. Yet, the agent-structure dichotomy might become less sharp in the long run. The reason is that the popularity of individual agentic action could motivate structural changes because political and business leaders cannot afford to be oblivious to public pressure. For example, the popularity of ESG (environmental, social, and corporate governance) investing (as opposed to shareholder wealth maximization) among major financial institutions might reflect a structural change in the financial sector in response to pressure from climate movements (Kotsantonis et al., 2016). Thus, instead of viewing them as substitutes, climate scholars should explore conditions under which agentic and structural approaches could co-evolve and complement each other.

In the next section, we explore the structure-agency debate in the context of low-carbon consumption and identify impediments to pro-climate choices and how these might be overcome. In the “Aviation emissions: structural and agentic approaches” section, we examine airline emissions from both the structural and agentic perspectives. In the concluding section, we draw lessons for climate action and outline themes for future research.

## Climate mitigation: structural and agentic approaches

Because climate change reflects the overuse of global commons, the climate discourse tends to focus on regulatory and business failures (a structural issue) as opposed to an absence of agentic “climate citizenship” (Wolf et al., 2009; Vihersalo, 2017). The landmark case *of Juliana v. United States* illustrates this argument. In this case, young plaintiffs contended that the US government had failed in its obligation to combat climate change. The plaintiffs argued that the atmosphere is a resource like air and water that the government is expected to hold in public trust. Consequently, the federal government has violated younger generations’ constitutional rights to life and liberty by not regulating its use. Importantly, the plaintiffs *did not* hold individual choices or consumption patterns accountable for the climate crisis: for them, the problem was structural, and therefore, blame was solely on the government.

But why have governments failed to regulate? Climate policies targeting fossil fuels impose an economic burden on specific sectors while everybody enjoys their benefits. In the absence of compensation for the sectors bearing mitigation costs (on the subject of “just transition,” see Newell and Mulvaney (2013)), these sectors and their allies have mobilized against mitigation policies. Moreover, governments have subsidized it instead of taxing the fossil fuel industry for the externalities it imposes on societies (Pigou, 1924). Thus, government failure is rooted in acts of omission (inadequate regulations) and commission (granting fossil fuel subsidies).

Regulating carbon emissions faces another challenge: the “China Excuse” (Dolšak and Prakash 2015). Because China is a non-Annex 1 country under the United Nations Framework Convention on Climate Change, it is not subject to mandatory emission reductions. Yet, the Chinese emissions are expanding, and since 2005, China has become the top carbon emitter. Ignoring the issue of historical responsibility, anti-climate actors see climate regulations as putting the US fossil fuel industry out of business, although China continues to build coal plants. As we discuss subsequently, the challenges in enacting new climate regulations probably underscore the need for consumer climate action which can take place independent of government policy.

Structures can be provided by businesses as well, especially when businesses voluntarily correct regulatory inaction (Matten and Moon, 2008)? Corporate social responsibility is on the agenda for modern corporations. Until recently, most companies did not embrace corporate climate responsibility, and some even funded the climate denial movement (Brulle, 2014). In recent years, however, many firms have adopted climate policies beyond their regulatory requirements. They have switched over to renewable energy, putting pressure on state governments to support renewable portfolio standards (Outka, 2019). A growing number of firms have also pledged to net-zero emissions by 2050. Yet, it is not clear if these commitments pertain to Scope 1 and 2 emissions (that result directly from the company’s activities or from electricity or heat that the company purchases from elsewhere) or if they also take into account Scope 3 emissions that pertain to their supply chain and consumers. The issue of the role of consumer choice, the agentic dimension to mitigation, is critical in addressing Scope 3 emissions.

Why do individuals not adopt low-emission lifestyles? Some consumers might have pro-climate preferences, but they might not recognize how their actions contribute to the climate crisis (Ropke, 2009). For Smith (1983), these consumers are “culpably ignorant” because they ought to connect the dots between consumption and emissions. Some consumers might recognize this link and yet believe that their actions alone cannot make a difference at the global level, the “causal inefficacy” hypothesis (Jamieson, 1992). Others might want to purchase low-carbon products but lack the tools to assess the carbon footprints of different products. “Carbon calculators” can help in this regard (Salo et al., 2019), but their proliferation might confuse when calculators generate different results for the same activity (Padgett, 2008).

Information can motivate behavioral changes when it is contextualized (Nerlich et al., 2010). A carbon calculator might reveal that the economy-class flight from London to New York creates almost 1 ton of CO_2_ emissions. This number might not mean much per se. However, if the passenger is also told that this is greater than the annual per capita emissions in 56 countries, this information might motivate pro-climate action, such as not flying at all or at least purchasing carbon offsets. Alongside carbon calculators, climate labels, such as Amazon’s Climate Pledge label, can provide climate information about specific products (Prakash and Potoski, 2006). Yet, consumers might be skeptical of climate labels due to greenwashing concerns (Wright and Nyberg, 2017). Thus, even motivated consumers wanting to adopt low-carbon lifestyles face considerable informational and cognitive hurdles.

Inter-connected consumption routines also impede pro-climate purchasing (Devinney et al., 2006). As “theories of practice” suggest, consumers may not evaluate the climate impact of individual actions in a piecemeal fashion (Salo et al., 2019). The reason is that individuals might be “locked-in” due to inter-connection among household activities. For example, an individual might want to stop using a car for office travel and switch to public transportation (Laakso, 2017). But this individual might also use a car to shop for groceries and to pick up children from their daycare. A switch to public transportation might reduce the carbon footprint of office commute but could jeopardize the person’s ability to perform household chores. Thus, climate policies might not lead to behavioral changes such as discontinuing driving unless policies can create system-level changes where this individual can rely on public transportation to travel to the office, do groceries, and pick up their children from daycare.

While government and business policies certainly affect how individuals exercise their consumption choices, could consumers influence structures that influence their choices? Fragnière (2016) suggests that consumers can do so collectively. Social movements have employed boycotts (and sometimes buycotts as well) to target firms and governments. Beck (2019) notes the cases of Irish peasants boycotting an English land agent, Charles Cunningham Boycott. Rev. Martin Luther King, Jr. organized the Montgomery bus boycott during the Civil Rights movement.

Under what conditions do boycotts influence the practices of the boycotted (Stolle and Micheletti, 2013)? For example, would businesses change practices only when the social movement pressure hurts their competitive position, profits, and stock prices (Pacheco and Dean, 2015)? Or would businesses react because they fear new regulations or the loss of legitimacy with stakeholders (Friedman, 1999)? As we discuss below, the “flight shame” movement is an example of consumer shaming targeted both at consumers (the direct target) and at the industry itself (the indirect target). By stigmatizing the act of flying (Cohen et al., 2011), it is shaping consumer demand and corporate behaviors and eventually contributing to new regulatory policies. Thus, social movement pressure might create pro-climate social norms that shape consumption choices and motivate regulatory and business initiatives to reduce aviation emissions.

In sum, both agentic and structural approaches to climate mitigation face considerable challenges. In the next section, we apply insights from the broader study of climate action to the specific case of aviation emissions.

## Aviation emissions: structural and agentic approaches

### Structural approaches to air travel emissions

Structural reasons contribute to the popularity of air travel. Scholars note that air travel is driven by income (“income effect”) and price (“substitution effect”) because demand for air travel has high income and price elasticities (Beckens and Carmignani, 2020). The substitution effect means that because airlines have dropped prices in the last two decades, individuals substitute air travel for other transportation modes, especially railways, which have a smaller carbon footprint.

But why have airlines dropped prices? Policy and institutional factors come into play here. Privatization and deregulation have led to the emergence of low-cost airlines (Clewlow et al. 2014), making air travel accessible to a larger number of people. This also means that along with a substitution effect, price declines increase real incomes and create an income effect as well.

Low ticket prices also result from government subsidies, especially on fuel. Since the 1944 Chicago Convention, which gave birth to the International Civil Aviation Organization (ICAO), governments are effectively prohibited from placing a value-added tax on international travel tickets (Havel and Sanchez, 2011). Yet, national, regional, and global policy changes are in motion — probably aided by social movement pressure on the airline industry. In 2012, the European Union (EU) began including emissions from intra-EU travel in the EU-Emission Trading Scheme (Scheelhaase et al., 2018). In recent years, Germany and France have enacted an aviation tax. France is proposing to ban flights where trains could cover the distance in less than 2.5 h. Although the ICAO has opposed new international rules to govern aviation emissions (Petersen, 2008), it has initiated measures to reduce the industry’s climate impact. In 2016, it rolled out CORSIA, the Carbon Offsetting and Reduction Scheme for International Aviation, to limit aviation’s “net emissions” to their average 2019–2020 levels. Due to the decline in air travel in response to COVID-19, there is a proposal to use only the 2019 emissions as the baseline.

While CORSIA is an industry-level response, airlines individually are also taking pro-climate steps. At one extreme, some airlines are taking the drastic step of “demarketing” (Kotler, 2011) by asking consumers to reduce flying. KLM’s “Fly Responsibly” campaign urges customers to consider alternative means of transportation, such as trains, for their short-haul travel needs (Hesse and Rünz, 2020). Others are taking less drastic measures, such as inducting modern low-emission aircraft. But most focus on carbon offsets. Alaska Airlines, Air Canada, Japan Airlines, and Cathay Pacific provide carbon calculators on their websites along with the opportunity for travelers to purchase carbon offsets. But other airlines purchase offsets themselves instead of expecting travelers to do so (Günther et al., 2020).

Carbon offsetting is an important tool in both structural and agentic approaches to tackle aviation emissions. The assumption is that air travel is a pillar of the modern economy and household lifestyles. Even with reduced travel and new technology such as biofuels, aviation emissions will continue. Thus, the industry will need to rely on carbon offsetting to reduce its climate impact. While scholars debate the morality and effectiveness of carbon offsetting (Foerster, 2019), it is used in several programs such as Clean Development Mechanisms, Reducing Emissions from Deforestation and Forest Degradation (REDD), and California’s Cap and Trade program (Lovell, 2010). For its supporters, the economic logic of offsetting is simple: actors should offset their climate impact instead of canceling these activities because activities generate sufficient value. However, critics point to the difficulty in verifying the climate benefits of offsetting. Scholars attribute travelers’ low take-up of voluntary carbon offsets offered by airlines to credibility problems (Zhang et al., 2019). Furthermore, offset programs such as fast-growing forests might inflict collateral damage on the ecosystem by, say, lowering the water table (Jackson et al., 2005). Finally, there is a danger that offset users might believe that they have a moral license to pollute and increase their consumption (Günther et al., 2020)

### Agentic approaches to air travel emissions

Individuals can contribute to climate mitigation in three inter-connected capacities: consumers, voters, and influencers. In all these roles, they may influence, intentionally or otherwise, the structures in which they are embedded. Foremost, as consumers, individuals seeking to do the “right thing” inadvertently support political or social issues, low-carbon consumption choices in our case. Scholars have called this “political consumerism” (Stolle and Micheletti, 2013) or “ethical consumerism” (Barry and MacDonald, 2018). Consequently, firms begin to see a potential for market demand for products with smaller carbon footprints (Roser-Renouf et al., 2016). Pro-climate consumers might also serve as activists and influencers seeking to change government and business policies. Individuals could participate in social movements to amplify the climate message embedded in consumption choices. Finally, as voters, individuals might support candidates with pro-climate agendas (De Moor & Verhaegen, 2020) who arguably could change policies influencing consumption choices. Voting efforts are hampered by collective action issues when individuals seek to free ride on the efforts of others or when they question the causal efficacy of their vote to shape electoral outcomes (Riker and Ordeshook, 1968). However, pro-climate consumption could foster a pro-climate “civic ethic” (Brennan, 2012), motivating individuals to overcome their reluctance to vote and help elect pro-climate candidates.

Agentic response to aviation emissions begins with individuals recognizing their culpability in creating them. While individuals with pro-environmental attitudes tend to support governmental action (Stoutenborough et al., 2014), would these individuals also curtail their aviation emissions? Alock et al. (2017) found no association between British respondents’ environmental concerns and their recreational flying decisions. Cocolas et al. (2020) find that individuals differentiate between the imperative to act on climate concerns when they are at home instead of taking a holiday. In sum, environmental concerns do not necessarily translate into reduced flying.

Why so? Sometimes individuals recognize carbon footprint issues but claim that flying is necessary for them. Higham et al. (2014: 462) call this the flyers’ dilemma: “the tension that exists between the perceived personal benefits of deeply embedded air travel practices and the collective climate change consequences of such practices.” Assuming actors are willing to overcome this dilemma, what should they do? At a minimum, they should reassess their decision to fly and explore alternative mechanisms to address the same task or need. For example, because the Coronavirus pandemic has “normalized” telecommuting for work-related tasks, might this motivate reduced flying in the future? Or individuals might decide to take holidays closer to home instead of flying to far-off destinations.

In the Internet age, individuals face little costs to search for information on the carbon footprint of air travel. There are several carbon calculators on the Web. Travel agencies and airlines provide carbon footprint information (along with offsetting options) when passengers search for tickets. Of course, policy innovations can support individual action. Even when policies do not dictate choices, they can help individuals make the right ones. Consider consumers seeking to buy airline tickets. Towards the end of the purchase, the travel website could inform them of their travel’s carbon footprint and ask if they want to buy offsets. Without this nudge, consumers may not even think of offsets. An even more aggressive strategy would be to make “opt-in” the default option. Here the travel website will automatically include carbon offsets in the ticket price unless the consumer opted out.

Individuals also play the important role of influencing others. For Fragnière (2016), individuals have a moral duty to serve as influencers irrespective of whether they believe that individual action is causally effective in reducing aggregate emissions. Individuals could do so through a bottom-up approach to change social norms or a top-down approach by lobbying governments and/or firms for more robust climate policies. Households might be more willing to adopt energy conservation measures or install solar panels when they learn that their neighbors have done so (Allcott and Rogers, 2014). Individuals could also influence their peers through social media and other forms of communication. Of course, not all individuals can aspire to be influencers. Moreover, even individuals with a large network of followers are more legitimate as climate champions when they lead a low-carbon lifestyle (Johnson, 2003; Hourdequin, 2010; Attari et al., 2019).

Individuals may also join a social movement working on reducing aviation emissions (Fragnière, 2016), for example, the Extinction Rebellion, Fridays for Future, and Flight Shame. These social movements might eventually encourage pro-climate practices and policy changes (Gössling, et al., 2020). As more people join a social movement, individuals might drop their skepticism about the causal efficacy of their actions, motivating higher levels of participation in the movement. As the movement gathers momentum and its “signal” crosses some sort of threshold (Bemmaor, 1984; Bolderdijk and Jans, 2021), both governments and businesses begin to pay close attention to the movement’s climate demands.

The role of the Flight Shame movement (*flygskam* in Swedish) is illustrative because it is creating an anti-flying social norm aimed at consumers, but with spillover effects on governments and the airline industry. Notes the contribution of the Instagram account Aningslösa Influencers (clueless influencers), with over 50,000 followers that shames Instagram members who brag about their air travel. Alongside stigmatizing a social practice, social movements can affirm pro-norm practices. For example, the case of “train brag” (*tågskryt* in Swedish) is critical in promoting rail travel.

What are the limitations of any social movement? Does the success of a movement depend on the structural context in which it functions? For example, the flight shame movement seems to be active predominantly in Europe. Is this because Europe has an excellent network of trains and shorter distances which make switching over from flying to trains easier in relation to, say, the USA? Others wonder if some social movements exaggerate problems and provoke overcorrection from individuals. For example, Chiambaretto et al. (2021) suggest that the “flight shame” movement creates a misleading narrative about the carbon footprint of air travel. Their study of French respondents found that 90% of respondents overestimate air transport’s share in global carbon emissions.

## Conclusion

**Fig. 1:**
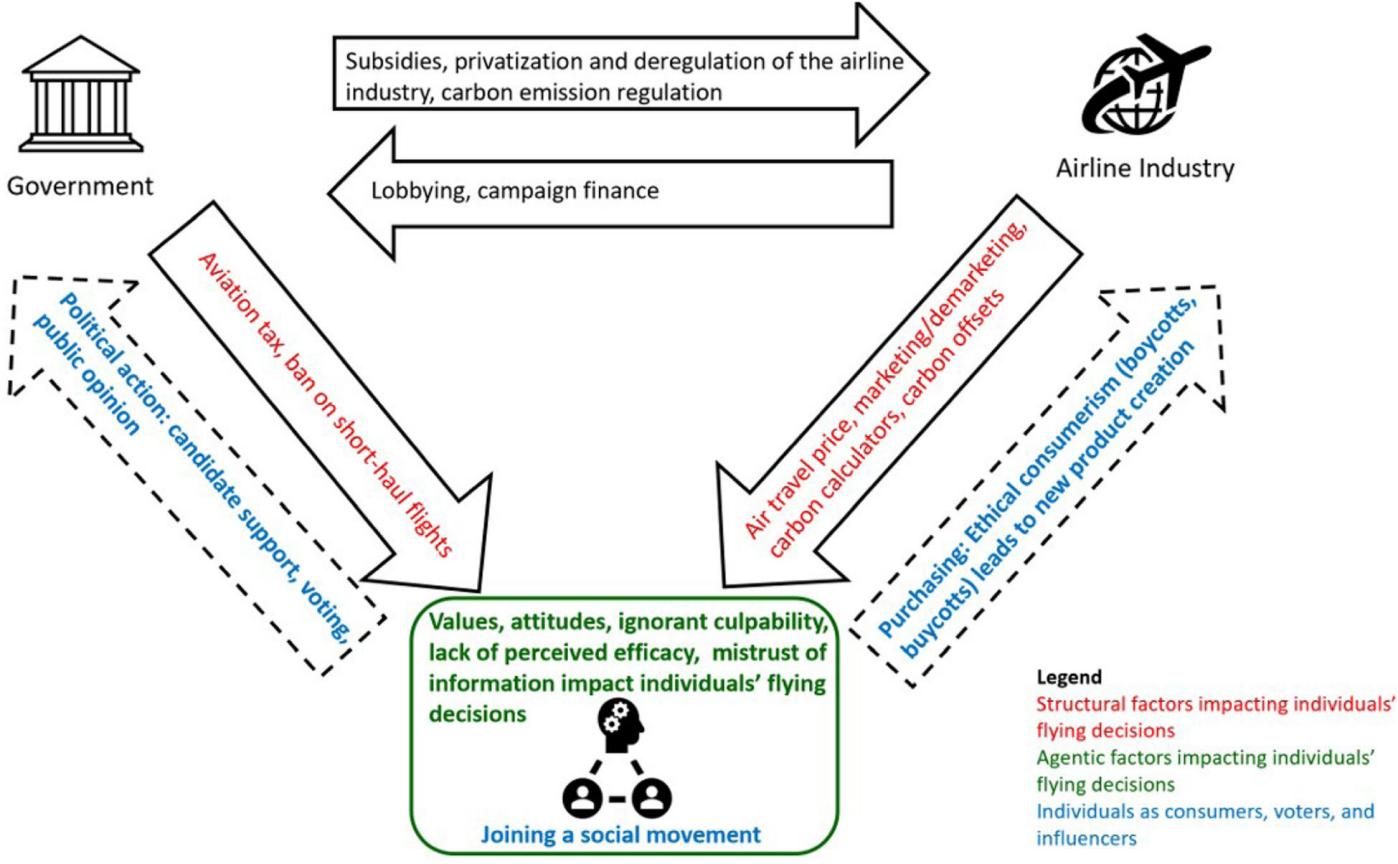
Structural and agentic approaches to reduce aviation emissions

In this review essay, we have examined agentic and structural approaches to tackling the complex issue of aviation emissions. Our framework is summarized in Fig. 1.

Future research should assess the conditions under which specific approaches are effective in the real world, as opposed to relying on projected reductions based on surveys. Indeed, we found little systematic evidence in the climate policy literature on this count. In part, since early 2020, COVID-19 has disrupted the airline and tourism industry, which has reduced air travel. This means that it is not clear whether reductions in air travel are due to agentic climate action, any specific climate policy governments have implemented, or individuals’ COVID-19 concerns. Once the COVID-19 pandemic is brought under control, scholars should assess the effectiveness of agentic and structural interventions in reducing aviation emissions.

The effectiveness of any approach depends on its political feasibility. Structural approaches are particularly vulnerable on this count. It is not clear if the governments have the political will to aggressively regulate aviation emissions, given the economic disruption such regulations might cause and its equity impacts. While much of the debate on “just transition” has focused on the fossil fuel sector, the airline industry will likely face the same challenges. Much to our surprise, we did not find much literature on this subject, although airlines and the airport ecosystems account for 20 million jobs (ATAG, 2016) and economies of many cities hosting major airports crucially depend on air travel (Conventz and Thierstein, 2014). Air travel is often crucial for the tourism industry, which supports another 30 million jobs. Future work should focus on just transition issues in the aviation sector and compare them with the debates in the fossil fuel industry.

Might agentic approaches face fewer political hurdles? From an agentic perspective, aviation emissions reflect carbon-intensive lifestyles. Although these are encouraged by rising incomes and government failure to regulate emissions, individuals have considerable autonomy to reduce their carbon footprint (Dietz et al., 2009). This is particularly true in airline travel, where individuals (in many cases) could satisfy the same need through lower-emission alternatives (such as trains or telecommuting), without facing a political pushback.

While agentic approaches might face fewer political hurdles, they require individuals to recognize their carbon footprints and act upon them. New policies can increase policy literacy and reduce search costs regarding carbon footprints, thereby motivating them to act. However, even policy-literate individuals might invoke causal inefficacy to justify their continued flying. Social pressure via social movements could create new social norms and motivate consumers to overcome the flyers’ dilemma.

The important lesson is that while individuals exercise autonomy in their consumption decisions and face fewer political hurdles in doing so, institutional innovation can facilitate pro-climate choices. Similarly, individuals can shape some of their embedded structures, especially when they coordinate their consumption choices via social movements. Viewed this way, the agent-structure dichotomy might be less sharp in the long term because under some conditions, agents and structures could exert reciprocal influence on each other (Archer, 1996).

What lessons might the airline industry hold for climate mitigation strategies in other sectors, especially in the context of agency-structure debate? Arguably, aviation is an easy case for agentic action because individuals often have choices regarding short-distance travel. Agency-focused approaches of the aviation sector could inform climate politics of the food, where individuals can exercise some level of agency, given the availability of cost-effective substitutes. The issue of reducing meat consumption, especially beef, has become salient in recent years. Individuals are participating in social movements to put pressure on restaurants to offer vegetarian options, including plant-based meat products. The menus of McDonald’s and Burger King include their version of “impossible burgers.” In response to student pressure, some educational institutions have stopped serving meat dishes in their cafeterias. The implication is that strong public pressure is bringing about change without the structural push in the form of public policy such as a meat tax or even a meat ban. On the other hand, agentic interventions might be less successful in the automobile industry. While individuals might be willing to buy electric vehicles (EVs), without a large-scale charging network, drives will face range anxiety and the EV uptake will suffer. Public policy interventions are necessary to establish a network of fast EV charging stations, given the complexity of addressing land use, zoning, and electricity grid regulations. Thus, the level of agentic autonomy to reduce emissions is industry-specific and lessons from the airline case need to be extended thoughtfully.
